# Supplementary material for: Reliability and validity of a newly developed Action Research Arm Test for upper limb function assessment in patients with stroke: A comparison with the conventional version
Source: PLoS One. 2026 Mar 24;21(3):e0334199. doi: 10.1371/journal.pone.0334199 (PMC13012481; doi:10.1371/journal.pone.0334199)
Supplement: S1 Protocol — (PDF) [file pone.0334199.s001.pdf]

Study Protocol

Title of Research: Verification of Clinical Usefulness of the Action Research Arm Test

Completion date: November 1, 2024

Ethics Applicant: Kensuke Taguchi
Principal Investigator: Daigo Sakamoto
Research Organization: Tokyo Jikei University Medical Hospital

1. Person in charge

1.1. Ethics Applicant

Kensuke Taguchi, Department of Rehabilitation Medicine, The Jikei University Medical Hospital

1.2. Principal Investigator

Daigo Sakamoto, Graduate School of Medicine, Jikei University

2. Purpose and Significance of the Study

2.1. Background and Objectives

Hemiplegia occurring in patients after stroke impairs activities of daily living and quality of life. Recovery of upper limb function is a critical goal of rehabilitation therapy. Effective rehabilitation interventions require accurate assessment tools to evaluate treatment outcomes. The Action Research Arm Test (ARAT) is the gold standard assessment tool for evaluating upper limb function in patients with stroke. The ARAT was developed by Lyle in 1981. It is an assessment method. It has excellent psychometric properties [1)]. In Japan, the ARAT has been widely adopted as the standard upper limb function assessment tool in both clinical and research settings.

However, the import of the conventional ARAT was discontinued, creating significant challenges for both clinical practice and research. Consequently, a domestically produced version of the instrument was developed. The new ARAT incorporates some modifications to the instrument specifications. While these changes may appear minor, findings from standardized assessment research indicate that even slight alterations in instrument characteristics can potentially affect task difficulty, movement strategies, and measurement precision [2)]. Introducing the modified instrument without rigorous psychometric validation risks undermining the continuity of research data and clinical practice standards established over decades by the conventional ARAT.

The purpose of this study is to establish the psychometric properties of the newly developed ARAT by assessing intra-rater reliability, inter-rater reliability, criterion-related validity against the conventional ARAT, and convergent validity with established clinical measures in patients with hemiplegia following stroke. This validation ensures a smooth transition from the conventional ARAT to the newly developed ARAT, maintaining measurement accuracy and clinical utility.

3. Methods

3.1. Research Design

This was a single-center, cross-sectional study.

3.2. Methods

3.2.1 Research Outline

In this study, participants will be assigned to a group for examiner reliability and inter-rater reliability (reliability validation group) and a group for criterion-related validity (validation group), and validation will be conducted according to the respective protocols.

・Reliability Verification Group

To verify intra-rater reliability, a single examiner performs two new ARATs at 15-minute intervals. During the initial assessment, the examiner will video record the subject's movements for all 19 tasks. To verify inter-rater reliability, a second examiner scores the video recordings without knowing the results of the first examiner's scoring.

・Validation group

A single examiner will administer both the new ARAT and the traditional version of the ARAT to verify criterion-relevant validity. The order of administration will be randomized using a random number table.

3.2.2. Dosage and administration of the study drug, duration of administration

Not applicable in this study, as no drug therapy was used.

3.2.3. Case Registration and Allocation Methods

Participants who meet eligibility criteria will be randomly assigned to either the reliability or validity validation group using a random number table. Group assignment and randomization of assessment order will be performed using a computer-generated random number sequence by the study coordinator, who will not be involved in data collection.

3.3. Outcome

3.3.1. Main outcome

・ARAT

3.3.2. Secondary outcome

・Fugl-Meyer Assessment of the Upper Extremity（FMA-UE）[3)]

・Box and Block Test (BBT) [4)]

・Motor Activity Log (MAL) [5)]

・Jikei Assessment Scale for Motor Impairment in Daily Living (JASMID) [6)]

3.4. Clinical characteristics of the patient

Basic information: sex, age, height, weight, Body Mass Index, dominant hand side before stroke onset

Medical information: stroke type, stroke onset area, stroke onset date, paralytic side

3.5 Statistical analysis

Participant characteristics, clinical assessment data: descriptive statistics

Intra-rater reliability: ICC (2,1)

Inter-rater reliability: ICC (2,1)

Measurement error: standard error of measurement (SEM)

Minimum detectable change: MDC95

Item-level agreement: weighted kappa coefficient with quadratic weighting.

Criterion-related validity: Bland-Altman analysis, Bland-Altman plots.

Convergent validity: Correlation analysis

Analyses were performed using Jamovi version 2.6.44 (https://www.jamovi.org). The statistical significance level is 5%.

4. Sample size and study period

4.1. Sample size

Total of 60 participants.

4.2. Basis for setting

The sample size for reliability verification was calculated based on prior research, assuming ICC = 0.8, minimum acceptable ICC = 0.6, α = 0.05, power (1 − β) = 0.8, two raters, and two measurements per participant [7,8]. This calculation indicated a required sample size of 28–30 participants. For criterion-related validity, G*Power 3.1 was used with an expected correlation coefficient r = 0.75, α = 0.05, and power = 0.95, yielding a minimum target sample size of 16 participants. Considering potential 10% data loss, 30 participants per group were planned.

4.3. Study period

The study is due to begin on 1 November 2024 and end on 31 May 2025.

5. Eligibility criteria for research participants

5.1. Criteria for selection

Participants will be recruited from patients who received occupational therapy at The Jikei University Medical Hospital during the study period. Participants will be those who meet all the following selection criteria and whose consent has been obtained.

(1) Age 18 or older
(2) Able to hold a sitting position independently
(3) Has hemiplegia after stroke

5.2. Exclusion Criteria

(1) impaired consciousness
(2) Cognitive impairment (Mini-Mental State Examination score of 25 or below) or a diagnosis of post-stroke cognitive impairment affecting the ability to understand instructions or perform tasks
(3) Recurrent stroke
(4) Visual field impairment
(5) Bilateral upper limb motor paralysis
(6) CNS or orthopedic disease other than stroke
(7) Upper limb or finger joint pain during movement
(8) Significant limitation of joint range of motion of the upper limb
(9) Amputation of upper limb, hand, or fingers
(10) Loss of data

6. Procedures for obtaining informed consent

6.1. Description of ethical considerations of the research

6.1.1. Regulatory Compliance

This research will be conducted in compliance with the "Declaration of Helsinki" and the "Ethical Guidelines for Life Sciences and Medical Research Involving Human Subjects (Ministry of Education, Culture, Sports, Science and Technology, Ministry of Health, Labor and Welfare, and Ministry of Economy, Trade and Industry, partially revised on March 10, 2022).

6.1.2. Compliance with research protocol

All investigators participating in this study will comply with this research protocol as long as it does not compromise the safety and human rights of patients.

6.1.3. Deviations from the research protocol

Researchers must not deviate from or change the research protocol before obtaining permission from the head of the research institution based on the Ethics Committee's prior review.

When the principal investigator becomes aware of a deviation or change, he/she shall promptly submit to the Ethics Committee the details and reasons of the deviation or change, and if a revision of the research protocol, etc. is necessary, a draft of the revision to the Ethics Committee and report it to the Ethics Committee and the head of the research institution. The principal investigator shall record all deviations from the research protocol together with the reasons for the deviation and the measures taken.

6.2. How to give informed consent

This study is a study that falls under the following.

1.	☐ Invasive (including minor invasion)<br>☑ Non-invasive
2.	☐ With intervention<br>☑ No intervention
3.	☐ (1) Use of newly acquired samples and information for research purposes<br>☑(2) Use only newly acquired information for research purposes<br>☐ (3) Use existing samples and information<br>☐ (4) Use only existing information
4.	☐ (1) There is transfer of samples and information to/from other institutions<br>☑(2) No transfer of samples/information to/from other institutions

This study utilizes information obtained within routine clinical practice; therefore, it will be conducted after obtaining written informed consent from research participants.

Researchers and others will provide the consent form approved by the ethics committee to the research participant (including a surrogate decision-maker when necessary; the same applies hereafter), provide sufficient explanation both verbally and in writing, and obtain written consent freely given by the research participant.

Researchers shall promptly provide information to research participants when information that could influence their consent is obtained, or when changes to the implementation plan or other aspects that could influence consent are made. They shall confirm the participants' intent regarding whether to participate in the research in advance.

Furthermore, they shall revise the consent explanation document and other materials after obtaining prior approval from the ethics committee and obtain renewed consent from the research participants.

7. Management of personal information

7.1. Handling of Personal Information

Researchers and heads of research institutions shall comply with the “Ethical Guidelines for Life Science and Medical Research Involving Human Subjects,” regulations and procedures established by the research institution, as well as the Personal Information Protection Act, ordinances, and other relevant laws and regulations regarding the handling of personal information, anonymized processed information, pseudonymized processed information, and personal-related information. Researchers shall appropriately handle personal information, etc., obtained while conducting research and held by the research institution to which the researcher belongs, to prevent leakage, loss, or damage and ensure other security management. The principal investigator shall, in cooperation with the head of the research institution, provide necessary guidance and management to other researchers, etc., handling such information to ensure the appropriate handling of held personal information, etc., during the conduct of research. When publishing the results of this research, researchers shall ensure that the information has been appropriately processed to prevent identification of specific individuals, thereby preventing any privacy-related disadvantage to research participants. Information that could identify specific individuals among the research participants involved in this research should not be shared with other research institutions or entities.

7.2. Method of processing in such a way that individuals cannot be identified

Researchers shall process the samples and information collected in this study by removing personal information such as names, initials, and patient IDs, then assigning new research IDs or numbers (hereinafter referred to as “research IDs, etc.”) for handling purposes, thereby rendering the data unidentifiable to specific individuals.
When processing data to prevent identification of specific individuals, researchers will create a table or record (hereinafter referred to as a “correspondence table”) linking personal information to research IDs or numbers. This correspondence table will enable the restoration of pseudonymized information (pseudonymized data) back to its original personal information.

The correspondence table is used, when necessary, such as when a research participant withdraws consent or declines participation, to restore pseudonymized information to personal information and dispose of the relevant research participant's samples and information. The correspondence table is managed under the responsibility of the principal investigator and stored in a locked cabinet within the research institution.

Furthermore, when publishing the results of this research, information that could identify specific research participants must not be included.

8. Anticipated risks and benefits

8.1. Expected profit

Participation in this study will not directly benefit the research participants. However, it is anticipated that by verifying the reliability and validity of the new ARAT, this research will contribute to rehabilitation treatment planning and goal setting, enabling patients to receive effective treatment.

8.2. Anticipated burdens and disadvantages

The examinations and assessments conducted in this study are routine clinical procedures and are non-invasive; therefore, participation is not expected to cause any burden or disadvantage.

8.3. Anticipated Risks

All upper limb function assessments conducted in this study are performed within the scope of routine clinical practice, and no new risks arise from this research. Patients may experience physical and mental fatigue or feel unwell due to examinations and evaluations. These are matters also observed during routine clinical care.

8.4. How to minimize risk

The examiner will conduct the assessment while observing whether participants appear excessively tense and inquiring about their physical and mental fatigue levels. During evaluation, appropriate rest periods will be scheduled as needed, taking care to minimize physical and mental strain as much as possible. If continuing the study becomes difficult, it will stop immediately. Should any participant report feeling unwell, this will be reported to the rehabilitation physician for appropriate examination and treatment

8.4.1. Criteria for discontinuation of individual participants

Research on a case shall be discontinued if any of the following discontinuation criteria are met.

(1) When a research participant voluntarily requests to withdraw their consent.

(2) When the principal investigator or other researcher determines that continuing the research is difficult due to worsening of the primary disease or complications.

(3) If a significant deviation from the research protocol is identified, such as a violation of ethical guidelines, inclusion criteria, or exclusion criteria.

(4) If compliance with the research protocol becomes impossible.

(5) If the entire study is discontinued.

(6) In other cases where the principal investigator or other researchers determine that continuation of the study is difficult.

[Basis for setting up]

These measures were established to ensure ethical research conduct and to safeguard the safety of research participants.

[How to respond in the event of cancellation]

The principal investigator and other researchers shall take appropriate measures for participants when discontinuing a study due to meeting discontinuation criteria, and clearly document the date and timing of discontinuation, reasons, and circumstances in medical records or similar documentation. Furthermore, if consent is withdrawn after the initiation of study treatment, the cause—whether due to an adverse event or an incidental event—shall be clarified as much as possible.

8.4.2. Criteria for discontinuation of the entire study

The principal investigator shall, after careful consideration, decide to terminate or suspend the research as necessary if any of the following conditions apply:

(1) When it is determined that patient recruitment is difficult and achieving the target number of participants is unlikely.

(2) When the research objectives are achieved before reaching the target number of participants or the planned research period.

(3) When the ethics committee recommends or orders termination.

9. Method of storage and disposal of samples and information

9.1. Storage of Samples

Since no samples are used in this study, this does not apply.

9.2. Storage of Information

9.2.1. Definition of information to be stored

The information subject to retention in this study is defined as copies of various application forms and reports, notifications from ethics committees and heads of research institutions, correspondence records, consent documents, copies of case report forms, records pertaining to information provision, and other documents or records necessary to ensure the reliability of data, including medical records.

9.2.2. Information storage method, storage period, and disposal method

The principal investigator shall retain the source materials defined in Section 10.2.1 for a period of five years from the date this research concludes, or until three years have elapsed from the date the final publication of this research's results was reported, whichever is later. However, at research institutions that stipulate a longer retention period, the materials shall be retained for the period specified in the institution's internal regulations.

Storage shall be conducted in lockable storage facilities. Other matters should be appropriately stored according to each institution's procedures. Furthermore, information and data collected in this research shall be anonymized by removing personal information such as names, initials, and patient IDs, assigning new research IDs, etc., to ensure specific individuals cannot be identified. This anonymized information shall then be appropriately stored by the records custodian at each institution. Correspondence tables shall be strictly managed under the responsibility of each institution's research principal investigator, with measures implemented to prevent leakage or loss.

When disposing of materials after the retention period ends, data stored on computers shall be erased using data erasure software. Paper documents shall be shredded with care to protect personal information. If separate procedures exist at each institution, those procedures shall be followed for appropriate disposal.

9.3. Transfer of Samples and Information with Other Institutions

This research does not involve the exchange of samples or information with other institutions and therefore does not apply.

10. Content and Method of Reporting to the Head of the Research Institution

1) When the Principal Investigator intends to conduct research (including cases where the research plan is modified), they shall prepare a research plan, obtain approval from the Ethics Committee, and then receive implementation permission from the head of the research institution. Furthermore, the Principal Investigator shall notify the research leader at the collaborating institution of the Ethics Committee's approval outcome, and the research leader at the

collaborating institution shall receive implementation permission from the head of their affiliated medical institution.
2) If a principal investigator obtains facts or information that compromise the ethical validity or scientific rationality of the research, or information that may compromise them and is deemed likely to affect the continuation of the research, they shall report this without delay to the head of the research institution and the principal investigator. The principal investigator shall, as necessary, suspend or terminate the research, or modify the research plan.
3) If the Principal Investigator obtains facts or information that undermine the appropriateness of the research implementation or the reliability of the research results, or information that may potentially undermine them, they shall promptly report this to the head of the research institution and the Principal Investigator. The Principal Investigator shall, as necessary, suspend or terminate the research, or modify the research plan.
4) The Principal Investigator (or Research Manager) shall, in principle, report annually to the head of the research institution on the progress of the research and the status of storage and management of acquired information.
5) Upon completion (including discontinuation; the same applies hereinafter) of the research, the Principal Investigator shall report to the head of the research institution and to the Research Manager of the collaborating research institution. The Research Manager of the collaborating research institution shall report to the head of their affiliated research institution.

11. Funding sources of research and conflicts of interest

11.1. Funding for research

This research is supported by a Grant-in-Aid for Scientific Research (Grant Number JP24K14384) from the Japan Society for the Promotion of Science (JSPS).

11.2. Conflicts of Interest and Personal Earnings, etc.

This research has no affiliation with any specific company or organization. In conducting this research, we strive to ensure transparency and impartiality. Researchers and others involved comply with their research institution's conflict of interest management regulations and have completed procedures with the committee responsible for managing conflicts of interest.

12. Method of disclosing information on research

Summary of this study and other matters Prior to implementation, register in the following public database. Update as appropriate in response to changes in the research plan and progress of the study. Upon completion of the study, register the results without delay.

Database for registration: University Hospital Medical Information Network Center (ID: UMIN000056693)

13. Handling of research results

This study does not apply because the evaluations and tests are conducted within routine clinical practice. However, once the study results are published, explanations will be provided upon request by research participants.

14. Responding to consultations, etc., from research participants and related parties

The contact point for questions and consultation regarding this study is as follows.

Department of Rehabilitation Medicine, The Jikei University Hospital

Daigo Sakamoto

Telephone number: 03-3433-1111 (ext. 3663)

15. Procedure for obtaining informed consent from a surrogate

This study excludes individuals under the age of 18 and those with severe cognitive impairment who are deemed unable to understand the explanation of research participation or provide informed consent. Therefore, the procedure for obtaining informed consent from a surrogate decision-maker does not apply and is not required.

16. Informed assent procedure

Participants in this study must be 18 years of age or older; therefore, they do not meet the inclusion criteria.

17. when the research is to be conducted under conditions of immediate and apparent life-threatening risk to the research participant

Not applicable to this study because the research is not expected to be conducted in an immediate and obvious life-threatening situation.

18. Financial burden and honorarium for research participants

18.1. Economic burden on research participants

The examinations and evaluations conducted in this study are performed within the scope of routine medical care; therefore, no additional costs will be incurred by the participants in this study.

18.2. Honorarium for research participants

In this study, no honorarium or burden reduction fee will be paid to study participants.

19. What to do in the event of a serious adverse event (for studies involving invasive procedures)

This study does not involve invasive procedures; therefore, the occurrence of serious adverse events is not anticipated.

20. Compensation for damage to health (in the case of research involving invasive procedures)

This study is not an interventional study; evaluations, examinations, and treatments are conducted within routine clinical practice. Therefore, since adverse health effects are not anticipated, it is not applicable.

21. Provision of post-study medical care to research participants

After the research is conducted, efforts will be made to ensure that research participants receive the best possible prevention, diagnosis, and treatment based on the results of the study.

22. Details of outsourced operations and method of supervising outsourced parties

This study does not involve outsourcing, so it is not applicable.

23. Secondary Use of Samples and Information Obtained from Research Participants

When obtaining consent from research participants, the intended purposes for using samples and information must be specified. If new purposes are subsequently identified, a research protocol must be created or amended and submitted for ethical review. Participants must then be notified of these new purposes, or the information must be made publicly available. Participants must be guaranteed an opportunity to withdraw their consent before the research proceeds.

24. Monitoring and auditing systems and procedures

24.1. Monitoring

This study is non-invasive and conducted within routine clinical practice; therefore, no monitoring will be performed.

24.2. Audit

This study is non-invasive and conducted within routine clinical practice; therefore, no audit will be performed.

25. References

1) Lyle RC. A performance test for assessment of upper limb function in physical rehabilitation treatment and research. Int J Rehabil Res. 1981;4(4):483–492.

2) Platz T, Pinkowski C, van Wijck F, Kim IH, di Bella P, Johnson G. Reliability and validity of arm function assessment with standardized guidelines for the Fugl-Meyer test, action research arm test and box and block test: a multicentre study. Clin Rehabil. 2005;19(4):404–411.

3) Fugl-Meyer AR, Jaasko L, Leyman I, Olsson S, Steglind S. The post-stroke hemiplegic patient. 1. a method for evaluation of physical performance. Scand J Rehabil Med. 1975;7(1):13–31.

4) Mathiowetz V, Volland G, Kashman N, Weber K. Adult norms for the Box and Block Test of manual dexterity. Am J Occup Ther. 1985;39(6):386–391. https://doi.org/10.5014/ajot.39.6.386

5) Uswatte G, Taub E, Morris D, Light K, Thompson PA. The Motor Activity Log-28: assessing daily use of the hemiparetic arm after stroke. Neurology. 2006;67(7):1189–1194.

6) Ishikawa A, Kakuda W, Taguchi K, Uruma G, Abo M. The reliability and validity of a new subjective assessment scale for poststroke upper limb hemiparesis, the Jikei assessment scale for motor impairment in daily living. Tokyo Jikei Med. J. 2010;125:159–167.
7) Walter SD, Eliasziw M, Donner A. Sample size and optimal designs for reliability studies. Stat Med. 1998;17(1):101–110.
8) Bujang MA. A simplified guide to determination of sample size requirements for estimating the value of intraclass correlation coefficient: a review. 2017;12(1):1-11.

Appendix 1. Handling of Personal Information at The Jikei University School of Medicine

1-1. Whether processing has been applied to prevent identification of individuals	☑ Perform processing<br>□ No processing<br>［Reason： ］
1-2. Availability of a table (correspondence table) that maps personal information to research IDs, etc.	☑ Create a correspondence table<br>□ Do not create a correspondence table<br>□ Correspondence sheets have been prepared outside of the University.<br>□ Already processed at the time of data acquisition<br>□ Other Reasons［ ］
1-3. Manager of correspondence table	Kensuke Taguchi<br>Tokyo Jikei University Medical Hospital
1-4. Correspondence table storage location	The Jikei University School of Medicine<br>Department of Rehabilitation Medicine Staff Room Lockable Cabinet
1-5. Personal Information protection consultation	Daigo Sakamoto<br>Department of Rehabilitation Medicine, The Jikei University Hospital<br>Telephone number: 03-3433-1111 (ext. 3663)
