# Supplementary material for: Reliability and validity of a newly developed Action Research Arm Test for upper limb function assessment in patients with stroke: A comparison with the conventional version
Source: PLoS One. 2026 Mar 24;21(3):e0334199. doi: 10.1371/journal.pone.0334199 (PMC13012481; doi:10.1371/journal.pone.0334199)
Supplement: S2 Protocol — (PDF) [file pone.0334199.s002.pdf]

研究実施計画書

研究課題名：Action Research Arm Test の臨床的有用性の検証

作成日：2024 年 11 月 1 日

研究代表者　：田口健介
担当者名　　：坂本大悟
研究機関　　：東京慈恵会医科大学附属病院

1.　研究の目的及び意義

1.1.　研究代表者

田口健介　東京慈恵会医科大学附属病院リハビリテーション科

1.2.　研究担当者

坂本大悟　東京慈恵会医科大学大学院医学研究科

2.　研究の目的及び意義

2.1.　背景と目的

脳卒中後の片麻痺は，患者の日常生活動作と生活の質を低下させる．上肢機能の回復は，重要なリハビリテーション治療の目標である．効果的なリハビリテーション介入には、治療効果を評価するための正確な評価ツールを要する．Action Research Arm Test（ARAT）は，脳卒中を有する患者に対する上肢機能評価のゴールドスタンダードの評価ツールである．ARATは1981年にLyleによって開発され，優れた心理測定特性を有する評価である[1)]．日本ではARATが臨床や研究場面において，標準的な上肢機能評価ツールとして広く採用されてきた．

しかし，従来型のARATの輸入が中止され，臨床・研究の両面で重大な課題を生じさせた．そのため，国産版の機器が開発された，新たなARATは機器仕様に一部変更を加えている．これらの変更は軽微に見えるが，標準化された評価研究の知見によれば機器特性のわずかな変化でさえ，課題難易度、動作戦略、測定精度に影響を及ぼし得る[2)]．厳密な心理測定学的検証を経ずに改良機器を導入することは，従来のARATが数十年にわたり確立してきた研究データと臨床実践基準の継続性を損なう恐れがある．

本研究の目的は，脳卒中後の片麻痺を有する患者を対象に，評価者内信頼性，評価者間信頼性，従来のARATに対する基準関連妥当性，および確立された臨床尺度との収束的妥当性を評価することにより，新たに開発されたARATの心理測定特性を確立することである．この検証により，従来のARATから新たなに開発されたARATへの円滑な移行が保証され，測定精度と臨床的有用性が維持される．

3.　研究の方法

3.1.　研究デザイン

本研究は単施設の横断研究である．

3.2.　研究方法

3.2.1　研究のアウトライン

本研究では，参加者を検査者信頼性および検査者間信頼性を検証する群（信頼性検証群）と基準関連妥当性を検証する群（妥当性検証群）に割り付けられ，各プロトコルに準じて検証が行われる．

・信頼性検証群

検査者内信頼性の検証のため，単一の検査者が新規 ARAT を 15 分間隔で 2 回実施する．初回評価時、検査者は全 19 課題の被験者動作をビデオ録画する．検査者間信頼性の検証のため，第 2 の検査者が第 1 検査者の採点結果を知らされない状態でビデオ記録を採点する

・妥当性検証群

基準関連妥当性の検証のため，単一の検査者が新規 ARAT と従来型 ARAT 版の両方を実施する．実施順序は乱数表を用いて無作為化する．

3.2.2.　試験薬の用法・用量，投与期間投与量・投与方法および投与期間等

本研究において，薬物治療は行われないため該当しない．

3.2.3.　症例登録・割付方法

適格基準を満たした参加者は，乱数表を用いて信頼性検証群または妥当性検証群のいずれかに無作為に割り付けられる．グループ割り付けと評価順序の無作為化は，データ収集に関与しない研究コーディネーターがコンピュータで作成した乱数列を用いて行う．

3.3.　評価項目

3.3.1.　主要評価項目

・ARAT

3.3.2.　副次的評価項

・Fugl-Meyer Assessment of the Upper Extremity（FMA-UE）[3)]
・Box and Block Test (BBT) [4)]
・Motor Activity Log (MAL) [5)]
・Jikei Assessment Scale for Motor Impairment in Daily Living (JASMID) [6)]

3.4.　患者情報

・基本情報：性別，年齢，身長，体重，Body Mass Index，発症前の利き手側
・医学的情報：脳卒中の病型，脳卒中の発症領，脳卒中の発症日，麻痺側

3.5.　統計解析

・参加者の特徴、臨床評価データ：記述統計
・評価者内信頼性：ICC（2,1）
・評価者間信頼性：ICC (2,1)
・測定誤差：標準誤差 (SEM)
・最小検出可能変化量：MDC_{95}
・項目レベルの一致：二次加重による加重カッパ係数.
・基準関連妥当性：Bland-Altman 分析，Bland-Altman プロット
・収束妥当性：相関分析

解析は Jamovi version 2.6.44 (https://www.jamovi.org)を用いて行う．統計学的有意水準は 5%とする．

4.　目標症例数及び予定期間

4.1.　目標症例数

合計 60 例（信頼性検証群 30 例，妥当性検証群 30 例）

4.2.　目標症例数の設定根拠

信頼性の検証のための最小目標症例数は、先行研究に基づき、ICC＝0.8，最小許容 ICC＝0.6，α＝0.05，検出力（1－β）＝0.8，評価者 2 名，参加者あたり測定 2 回を仮定して算出された[7,8]．この計算により，28〜30名の参加者が必要とされた．基準関連妥当性については、G*Power 3.1 を用い，期待相関係数 r = 0.75、α = 0.05、検出力 = 0.95 を設定した結果，最小目標症例数は 16 名と算出された．潜在的な 10%のデータ損失を考慮し，各群 30 名を対象とする．

4.3.　研究実施予定期間

研究実施予定期間：2024 年 11 月 1 日から 2025 年 3 月 31 日まで

5.　研究参加者の選定方針

5.1.　選択基準

参加者は研究期間中に東京慈恵会医科大学附属病院において作業療法が実施された患者から募集を行う．以下の選択基準を全て満たし，且つ本人から同意を得られた者を参加者とする．

(1) 年齢が18歳以上

(2) 自立した座位の保持が可能

(3) 脳卒中後の片麻痺を有するもの

5.2.　除外基準

(1) 意識障害

(2) 認知機能障害（Mini-Mental State Examination スコアが 25 点以下）または指示の理解や作業遂行能力に影響を及ぼす脳卒中後の認知機能障害の診断

(3) 脳卒中の再発

(4) 視野障害

(5) 両側上肢運動麻痺

(6) 脳卒中以外の中枢神経系または整形外科疾患

(7) 運動時の上肢または指関節の痛み

(8) 上肢の関節可動域の著しい制限

(9) 上肢、手、指の切断

(10) データの欠損

6.　インフォームド・コンセントを受ける手続等

6.1.　研究の倫理的配慮に関する記述

6.1.1.　規制の遵守

本研究は「ヘルシンキ宣言」および「人を対象とする生命科学・医学系研究に関する倫理指針（文部科学省・厚生労働省・経済産業省　令和4年3月10日一部改正）」を遵守して研究を実施する．

6.1.2.　研究計画書の遵守

本研究に参加する研究者は，患者の安全と人権を損なわない限り，本研究計画書を遵守する.

6.1.3.　研究実施計画書からの逸脱

研究者等は，倫理委員会の事前の審査に基づく研究機関の長の許可を得る前に，研究実施計画書からの逸脱あるいは変更を行ってはならない.

研究責任者は，逸脱あるいは変更の事実を知ったときは，逸脱または変更の内容および理由ならびに研究計画書等の改訂が必要であればその案を速やかに，倫理委員会に提出し，倫理委員会および研究機関の長に報告を行う．研究責任者は，研究計画書からの逸脱があった場合は逸脱事項をその理由および措置とともに全て記録する.

6.2.　インフォームド・コンセントの方法

本研究は，以下に該当する研究である.

1.	☐侵襲あり（軽微な侵襲を含む）<br>☑侵襲なし
2.	☐介入あり<br>☑介入なし
3.	□(1)研究目的で新たに取得した試料と情報を使用する<br>☑(2)研究目的で新たに取得した情報のみを使用する<br>□(3)既存試料と情報を使用する<br>□(4)既存情報のみを使用する
4.	□(1)他機関と試料・情報の授受がある<br>☑(2)他機関と試料・情報の授受がない

本研究は，通常診療内で取得される情報を使用するため，研究参加者より文書によりインフォームド・コンセントを得た上で研究を実施する.

研究者等は，倫理委員会で承認の得られた同意説明文書を研究参加者（代諾者が必要な場合は代諾者を含む，以下に同じ）に渡し，文書および口頭による十分な説明を行い，研究参加者の自由意思による同意を文書で取得する.

研究者等は，研究参加者の同意に影響を及ぼす情報が得られたときや，研究参加者の同意に影響を及ぼすような実施計画等の変更が行われるときは，速やかに研究参加者に情報を提供し，研究に参加するか否かについて研究参加者の意思を予め確認するとともに，事前に倫理委員会の承認を得て同意説明文書等の改訂を行い，研究参加者の再同意を得ることとする.

7.　個人情報の管理

7.1.　個人情報等の取扱い

研究者等及び研究機関の長は，個人情報，匿名加工情報及び仮名加工情報及び個人関連情報の取扱いに関して，「人を対象とする生命科学・医学系研究に関する倫理指針」や研究機関の定める規定・手順書のほか，個人情報保護法，条例等を遵守する.

研究者等は，研究の実施に伴って取得された個人情報等であって当該研究者等の所属する研究機関が保有

しているものについて，漏えい，滅失又はき損の防止その他の安全管理のため，適切に取り扱う.
研究責任者は，研究の実施に際して，保有する個人情報等が適切に取り扱われるよう，研究機関の長と協力しつつ，当該情報を取り扱う他の研究者等に対して，必要な指導・管理を行う.
本研究の結果を公表する際は，研究参加者にプライバシー上の不利益が生じないよう，適切に特定の個人を識別できないように加工されていることを確認した上で公表を行う.
本研究に係る研究参加者の特定の個人を識別できる情報は，他の研究機関等と共有しない.

7.2. 個人を識別できないように加工する方法

研究者等は，本研究で収集する試料・情報から，氏名，イニシャル，患者 ID などの個人情報を削除した上で，研究のための ID あるいは番号（以下，研究 ID 等）を新たに付与して取扱い，特定の個人を識別できないように加工する.

特定の個人を識別できないように加工する際は，個人情報と研究用 ID・番号を対応させる表や記録（以下，対応表）を作成して，特定の個人を識別できないように加工された情報（仮名加工情報）を個人情報に復元できるようにする.

対応表は，研究参加者からの同意撤回や参加拒否の申し出があった場合など，必要に応じて仮名加工情報を個人情報に復元し，該当の研究参加者の試料や情報を破棄する際に用いられる．対応表の管理は，研究責任者の責任の下，研究機関内の施錠されたキャビネット内で保管される.

なお，本研究の結果を公表する際は，特定の研究参加者を識別できる情報を含まないようにする.

8. 予測されるリスク及び利益

8.1. 予測される利益

本研究へ参加することによる研究参加者に直接の利益は生じない．しかし，本研究により新たな ARAT の信頼性および妥当性が検証されることにより，リハビリテーションの治療計画や目標設定に役立てることができ，患者は効果的な治療を享受できるようになると推察される.

8.2. 予測される負担・不利益

本研究で行われる検査，評価は通常診療内で行われているものであり，侵襲を伴わないため，参加することにより予測される負担・不利益はない.

8.3. 予測されるリスク

本研究で行われる上肢機能評価は全て通常の診療内で行われていることであり，本研究により生じる新たなリスクはない．患者には，検査，評価によって身体的及び精神的な疲労，体調不良等が生じる可能性がある．これは，通常診療のなかでも観察される事項である.

8.4. リスクを最小化する方法

参加者が過度に緊張している様子がないかを観察し，身体的，精神的疲労の状態について問診を行いながら，実施する．また評価中は，適宜休息時間を設定することとし，可能な限り身体的，精神的負担を生じさせないように留意する．研究の続行が困難となった際には，ただちに中止する．体調不良等の訴えがあった場合は，リハビリテーション科医師に報告し，適切な診察と治療を行う.

8.4.1.　個々の参加者の中止基準

以下の中止基準に該当する場合は，当該症例の研究を中止する.

(1) 研究参加者の自由意思による同意撤回の申し入れがあった場合.
(2) 研究責任者又は研究者等により，原疾患あるいは合併症の悪化のため，研究の継続が困難と判断された場合.
(3) 倫理指針，選択基準違反又は除外基準違反など研究計画書からの重大な逸脱が判明した場合
(4) 研究計画書の遵守が不可能になった場合
(5) 研究全体が中止された場合
(6) その他，研究責任者又は研究者等により研究の継続が困難と判断された場合

【設定根拠】

研究を倫理的に実施するため，また，研究参加者の安全性に配慮して設定した.

【中止時の対応方法】

研究責任者及び研究者等は，中止基準に該当するため研究を中止した場合には，当該研究の参加者に対し適切な措置を講じ，中止の日付・時期，理由，経過等を診療記録等に明記する．なお，研究治療開始後に同意の撤回があった場合は，その原因が有害事象等によるものか，あるいは偶発的事象（転居など）によるものかをできるだけ明らかにする.

8.4.2.　研究全体の中止基準

研究責任者は，以下の事項に該当する場合は，検討の上，必要に応じて，研究の中止・中断を決定する.

(1) 患者のリクルートが困難で目標参加者数の登録達成が困難であると判断した場合
(2) 目標参加者数または研究実施予定期間に達する前に研究の目的が達成された場合
(3) 倫理委員会により中止の勧告または指示があった場合

9.　試料・情報の保管及び廃棄の方法

9.1.　試料の保管について

本研究では試料は用いないため，該当しない.

9.2.　情報の保管について

9.2.1.　保管する情報の定義

本研究において保管対象となる情報は，各種申請書・報告書の控え，倫理委員会・研究機関の長からの通知書，対応表，同意文書，症例報告書等の控え，情報の提供に関する記録，その他診療記録を含むデータの信頼性を保証するのに必要な書類または記録と定義する.

9.2.2.　情報の保管方法，保管期間，廃棄方法

研究責任者は，10.2.1.で定義した原資料を，本研究が終了した日から5年間又は本研究の結果の最終の公表について報告された日から3年を経過した日のいずれか遅い日までの期間保存する．但し，この期間を超

えた保管期間を定めている研究機関においては，機関内の規定に則った期間保存する．
なお，保存は，施錠可能な保管庫で行うものとし，その他事項は各機関の手順に従い適切に保存する．また，本研究で収集する情報・データは，氏名，イニシャル，患者IDなどの個人情報を削除し，新たに研究ID等をつけ，特定の個人が識別できないように仮名加工情報にした上で，各機関の記録保管責任者が適切に保管する．なお，対応表は各機関の研究責任者の責任の下，漏えいや紛失の防止策を講じた上で厳重に管理する．

保存期間終了後に廃棄する場合は，パソコン内のデータについては消去ソフトを使用して消去し，また紙資料については個人情報に注意しシュレッダーで裁断する他，別途各機関に手順がある場合はその手順に従い適切に廃棄する．

9.3.　他機関との試料・情報の授受について

本研究は，他機関と試料・情報の授受がないため，該当しない．

10.　研究機関の長への報告内容及び方法

1）研究代表者は，研究を実施（研究計画書を変更して実施する場合を含む）しようとするときは，あらかじめ研究計画書を作成し，倫理委員会の承認を得た上で，研究機関の長の実施許可を受ける．また，研究代表者は倫理委員会の承認結果を共同研究期間の研究責任者に通知し，共同研究機関の研究責任者は所属する医療機関の長の実施許可を受ける．

2）研究責任者は，研究の倫理的妥当性もしくは科学的合理性を損なう事実もしくは情報又は損なうおそれのある情報であって研究の継続に影響を与えると考えられるものを得た場合には，遅滞なく，研究機関の長及び研究代表者に対して報告し，研究代表者は必要に応じて研究を停止し，若しくは中止し，又は研究計画書を変更する．

3）研究責任者は，研究の実施の適正性もしくは研究結果の信頼を損なう事実もしくは情報又は損なうおそれのある情報を得た場合には，速やかに研究機関の長及び研究代表者に報告し，研究代表者は必要に応じて研究を停止し，もしくは中止し，又は研究計画書を変更する．

4）研究代表者（または研究責任者）は，原則として年に1回，研究の進捗状況及び取得された情報等の保管・管理状況について研究機関の長に報告する．

5）研究代表者は，研究を終了（中止の場合を含む．以下同じ．）したときは，研究機関の長ならびに共同研究機関の研究責任者に報告する．共同研究機関の研究責任者は所属する研究機関の長に報告する．

11.　研究の資金源と利益相反

11.1.　研究の資金源

この研究はJSPS科研費 JP24K14384の助成により実施される．

11.2.　利益相反および個人の収益等

この研究は，特定の企業・団体との関与はない．研究の実施に当たり，研究の透明性および公平性の確保

に努める．なお，研究者等は研究機関の利益相反管理に関する規定を遵守し，利益相反を管理する委員会への手続きを行っている．

12.　研究に関する情報公開の方法

本研究の概要その他実施に先立って以下の公開データベースに登録し，研究計画書の変更及び研究の進捗に応じて適宜更新し，研究を終了したときは，遅滞なく結果を登録する．
登録を行うデータベース：大学病院医療情報ネットワークセンター（ID: UMIN000056693）

13.　研究結果等の取扱い

本研究は通常診療内で行われる評価や検査であるため，該当しない．但し，研究の成果が公表された後は，研究参加者の求めがあれば説明を行う．

14.　研究参加者及びその関係者からの相談等への対応

本研究に関する質問および相談の窓口は以下の通りである．
研究機関名・部署：東京慈恵会医科大学附属病院・リハビリテーション科
担当者：坂本大悟
電話番号（内線）: 03-3433-1111（内線 3663）

15.　代諾者等からインフォームド・コンセントを受ける手続

本研究では，20歳に満たないもの，また研究への参加に関する説明の理解及び同意が困難と考えられる重度の認知機能障害を呈するものは対象には加えない．よって，代諾者からインフォームド・コンセントを受ける手続きは発生しないため，該当しない

16.　インフォームド・アセントの手続

本研究の参加者の取り込み基準は，年齢が20歳以上であるため，該当しない．

17.　研究参加者に緊急かつ明白な生命の危機が生じている状況で研究を実施しようとする場合

本研究では，緊急かつ明白な生命の危機が生じている状況において研究を実施することは想定されないため，該当しない．

18.　研究参加者の経済的負担および謝礼

18.1.　研究参加者の経済的負担

本研究で行われる検査，評価は通常診療内で行われるため，本研究の参加者に新たな費用負担は発生しない．

18.2.　研究参加者への謝礼

本研究では，研究参加者に謝礼や負担軽減費等の支払いは行わない．

19.　重篤な有害事象が発生した際の対応（侵襲を伴う研究の場合）

本研究は，侵襲を伴う研究に該当しないため，重篤な有害事象の発生は想定されない.

20.　健康被害に対する補償（侵襲を伴う研究の場合）

本研究は観察研究であり，評価，検査，治療は通常診療内で行われる．よって，健康被害の発生が想定されないため，該当しない.

21.　研究参加者への研究実施後における医療の提供（介入を行う研究の場合）

研究実施後，研究参加者が当該研究の結果により得られた最善の予防，診断及び治療を受けることができるよう努める.

22.　委託業務内容及び委託先の監督方法（業務委託をする場合）

本研究では業務委託を行わないため，該当しない.

23.　研究参加者から取得された試料・情報の二次利用について

研究参加者らから同意を受ける時点で想定される試料・情報の利用目的等について，その後，利用目的等が新たに特定されたときは，研究計画書を作成又は変更し倫理審査を受けた上で，新たに特定された利用目的等についての情報を研究参加者に通知し，又は公開し，研究が実施されることについて，研究参加者らが同意を撤回できる機会を保障する.

24.　モニタリング及び監査の実施体制及び実施手順（侵襲・介入を伴う研究の場合）

25.1.　モニタリング

本研究は侵襲を伴わず，通常診療内で行われる研究であるため，モニタリングは実施しない.

25.2.　監査

本研究は侵襲を伴わず，通常診療内で行われる研究であるため，監査は実施しない.

25.　参考文献

1) Lyle RC. A performance test for assessment of upper limb function in physical rehabilitation treatment and research. Int J Rehabil Res. 1981;4(4):483–492.

2) Platz T, Pinkowski C, van Wijck F, Kim IH, di Bella P, Johnson G. Reliability and validity of arm function assessment with standardized guidelines for the Fugl-Meyer test, action research arm test and box and block test: a multicentre study. Clin Rehabil. 2005;19(4):404–411.

3) Fugl-Meyer AR, Jaasko L, Leyman I, Olsson S, Steglind S. The post-stroke hemiplegic patient. 1. a method for evaluation of physical performance. Scand J Rehabil Med. 1975;7(1):13–31.

4) Mathiowetz V, Volland G, Kashman N, Weber K. Adult norms for the Box and Block Test of manual dexterity. Am J Occup Ther. 1985;39(6):386–391. https://doi.org/10.5014/ajot.39.6.386

5) Uswatte G, Taub E, Morris D, Light K, Thompson PA. The Motor Activity Log-28: assessing daily use of the hemiparetic arm after stroke. Neurology. 2006;67(7):1189–1194.

6) Ishikawa A, Kakuda W, Taguchi K, Uruma G, Abo M. The reliability and validity of a new subjective assessment scale for poststroke upper limb hemiparesis, the Jikei assessment scale for motor impairment in daily living. Tokyo Jikei Med. J. 2010;125:159–167.

7) Walter SD, Eliasziw M, Donner A. Sample size and optimal designs for reliability studies. Stat Med. 1998;17(1):101–110.

8) Bujang MA. A simplified guide to determination of sample size requirements for estimating the value of intraclass correlation coefficient: a review. 2017;12(1):1-11.

別添 1　東京慈恵会医科大学における個人情報の取扱い

1-1.個人を識別できないようにする加工の有無	☑加工を行う<br>□加工を行わない<br>［理由：　　］
1-2.個人情報と研究 ID 等を対応させる表（対応表）の有無	☑対応表を作成する<br>□対応表を作成しない<br>□本学以外で対応表が作成されている（本学では加工された試料・情報のみを取り扱う）<br>□データ取得時点で既に加工されている（過去の研究の二次利用や無記名のアンケートの場合など）<br>□その他［詳細：　　］
1-3.対応表の管理者（本研究における本学の個人情報管理者）	所属：東京慈恵会医科大学医学研究科　氏名：坂本大悟
1-4.対応表の保管場所	東京慈恵会医科大学<br>リハビリテーション医学講座 スタッフルーム　鍵付きキャビネット
1-5.個人情報保護相談窓口	坂本大悟<br>東京慈恵会医科大学附属病院：03-3433-1111（内線 3661）<br>対応時間：午前９時～午後４時／休診日を除く
