# Supplementary material for: Reliability and validity of a newly developed Action Research Arm Test for upper limb function assessment in patients with stroke: A comparison with the conventional version
Source: PLoS One. 2026 Mar 24;21(3):e0334199. doi: 10.1371/journal.pone.0334199 (PMC13012481; doi:10.1371/journal.pone.0334199)
Supplement: S3 Checklist — (PDF) [file pone.0334199.s003.pdf]

The CONSORT reporting checklist

For checking that reports of randomised trials can be understood and used by everyone

ⓘ Note

If you have not used a reporting guideline before, read about how and why to use them and check whether CONSORT is the most applicable reporting guideline for your work.

Reporting guidelines are most useful when used early in research. When writing a manuscript or application, consider using the Full Guidance where you'll see explanations and examples for each item.

After writing, demonstrate adherence by completing this checklist:

1. Specify where each item is described (see Note 1).
2. Cite this checklist (See Note 2).
3. Include your completed checklist as a supplement when submitting to a journal so that future readers can use it to find information.

	Item Description	Location (or reason for not reporting)
**Title and Abstract**		
1a. Title	Identification as a randomised trial.	N/A<br>This study employed a cross-sectional observational study design.
1b. Structured Abstract	Structured summary of the trial design, methods, results, and conclusions.	p2, Abstract
**Open Science**		
2. Trial Registration	Name of trial registry, identifying number (with URL) and date of registration.	p5, Materials and Methods, Ethical Considerations
Protocol and statistical analysis plan	Where the trial protocol and statistical analysis plan can be accessed.	This is not included in this manuscript.
4. Data sharing	Where and how the individual de-identified participant data (including data dictionary), statistical code and any other materials can be accessed.	p24, Availability of Data and Materials
5. Funding and Conflicts of Interest		
5a. Funding	Sources of funding and other support (eg, supply of drugs), and role of funders in the design, conduct, analysis, and reporting of the trial.	This is not included in this manuscript. This point has been reported to the submission system.

5b. Conflicts of interest	Financial and other conflicts of interest of the manuscript authors.	This is not included in this manuscript. This point has been reported to the submission system.
**Introduction**		
6. Background and rationale	Scientific background and rationale.	p3-5, Introduction; paragraphs 1-7
7. Objectives	Specific objectives related to benefits and harms.	p5, Introduction; paragraph 8
**Methods**		
8. Patient and public involvement	Details of patient or public involvement in the design, conduct and reporting of the trial.	p5, Materials and Methods, Study Design
9. Trial Design	Description of trial design including type of trial (eg, parallel group, crossover), allocation ratio, and framework (eg, superiority, equivalence, non-inferiority, exploratory).	p5, Materials and Methods, Study Design
10. Changes to trial protocol	Important changes to the trial after it commenced including any outcomes or analyses that were not pre-specified, with reason.	N/A
11. Trial Setting	Settings (eg, community, hospital) and locations (eg, countries, sites) where the trial was conducted.	p7, Materials and Methods, Setting
12. Eligibility Criteria		
12a. Participants	Eligibility criteria for participants.	p6, Materials and Methods, Participants; paragraphs 1-2
12b. Other	If applicable, eligibility criteria for sites and for individuals delivering the interventions (eg, surgeons, physiotherapists).	p10, Materials and Methods, Experimental Procedures, Investigators
13. Intervention and comparator	Intervention and comparator with sufficient details to allow replication. If relevant, where additional materials describing the intervention and comparator (eg, intervention manual) can be accessed.	N/A<br>This study employed a cross-sectional observational study design.
14. Outcomes	Prespecified primary and secondary outcomes, including the specific measurement variable (eg, systolic blood pressure), analysis metric (eg, change from baseline, final value, time to event), method of aggregation (eg, median, proportion), and time point for each outcome.	p9, Materials and Methods, Experimental Procedures, Clinical evaluations and Participant characteristics
15. Harms	How harms were defined and assessed (eg, systematically, non-systematically).	N/A
16. Sample Size		
16a. How sample size was determined	How sample size was determined, including all assumptions supporting the sample size calculation.	p6, Materials and Methods, Sample Size Calculation

16b. Interim analyses and stopping criteria	Explanation of any interim analyses and stopping guidelines.	N/A
17. Randomisation		
17a. Sequence Generation	Who generated the random allocation sequence and the method used.	p6-7, Materials and Methods, Randomization and Blinding
17b. Type of Randomisation	Type of randomisation and details of any restriction (eg, stratification, blocking, and block size).	p6-7, Materials and Methods, Randomization and Blinding
18. Allocation concealment mechanism	Mechanism used to implement the random allocation sequence (eg, central computer/telephone; sequentially numbered, opaque, sealed containers), describing any steps to conceal the sequence until interventions were assigned.	p6-7, Materials and Methods, Randomization and Blinding
19. Implementation	Whether the personnel who enrolled and those who assigned participants to the interventions had access to the random allocation sequence.	This is not included in this manuscript.
20. Blinding		
20a. Who was blinded	Who was blinded after assignment to interventions (eg, participants, care providers, outcome assessors, data analysts).	p6-7, Materials and Methods, Randomization and Blinding
20b. How blinding was achieved	If blinded, how blinding was achieved and description of the similarity of interventions.	p6-7, Materials and Methods, Randomization and Blinding
21. Statistical methods		
21a. Comparing groups	Statistical methods used to compare groups for primary and secondary outcomes, including harms.	p10-11, Materials and Methods, Statistical Analysis
21b. Definition of who is included in each analysis	Definition of who is included in each analysis (e.g., all randomised participants), and in which group.	p10-11, Materials and Methods, Statistical Analysis
21c. Missing Data	How missing data were handled in the analysis.	p6, Materials and Methods, Participants; paragraph 2
21d. Additional Analyses	Methods for any additional analyses (eg, subgroup and sensitivity analyses), distinguishing pre-specified from post hoc.	N/A
22. Participant flow, including flow diagram		
22a. Participant Numbers	For each group, the numbers of participants who were randomly assigned, received intended intervention, and were analysed for the primary outcome.	p12, Results, Fig 1

22b. Losses and exclusions	For each group, losses and exclusions after randomisation, together with reasons.	p12, Results; paragraph 1
23. Recruitment		
23a. Dates	Dates defining the periods of recruitment and follow-up for outcomes of benefits and harms.	N/A
23b. Reasons for stopping recruitment	If relevant, why the trial ended or was stopped.	N/A
24. Intervention and comparator delivery		
24a. As Administered	Intervention and comparator as they were actually administered (eg, where appropriate, who delivered the intervention/comparator, whether participants adhered, whether they were delivered as intended (fidelity)).	This is not included in this manuscript. This study employed a cross-sectional observational study design.
24b. Concomitant Care	Concomitant care received during the trial for each group.	N/A
25. Baseline Data	A table showing baseline demographic and clinical characteristics for each group.	p12-14, Results; paragraph 2, Table 1 and 2
26. Numbers analysed, outcomes, and estimation	For each primary and secondary outcome, by group:<br>• the number of participants included in the analysis.<br>• the number of participants with available data at the outcome time point.<br>• result for each group, and the estimated effect size and its precision (such as 95% confidence interval).<br>• for binary outcomes, presentation of both absolute and relative effect size.	p14-21, Results; paragraphs 3-7
27. Harms	All harms or unintended events in each group.	N/A
28. Ancillary Analyses	Any other analyses performed, including subgroup and sensitivity analyses, distinguishing pre-specified from post hoc.	N/A
**Discussion**		
29. Interpretation	Interpretation consistent with results, balancing benefits and harms, and considering other relevant evidence.	p22-23, Discussion; paragraphs 1-4
30. Limitations	Trial limitations, addressing sources of potential bias, imprecision, generalisability, and, if relevant, multiplicity of analyses.	p23, Discussion; paragraph 5
