# Supplementary figures and images for: Reliability and validity of a newly developed Action Research Arm Test for upper limb function assessment in patients with stroke: A comparison with the conventional version
Source: PLoS One. 2026 Mar 24;21(3):e0334199. doi: 10.1371/journal.pone.0334199 (PMC13012481; doi:10.1371/journal.pone.0334199)

(a)

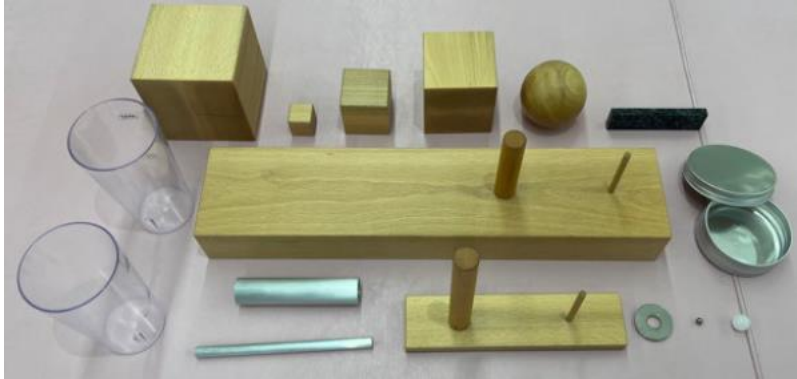

(b)

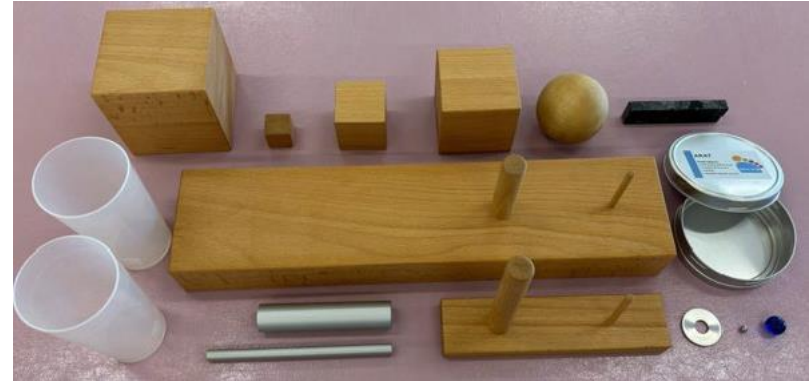

(c)

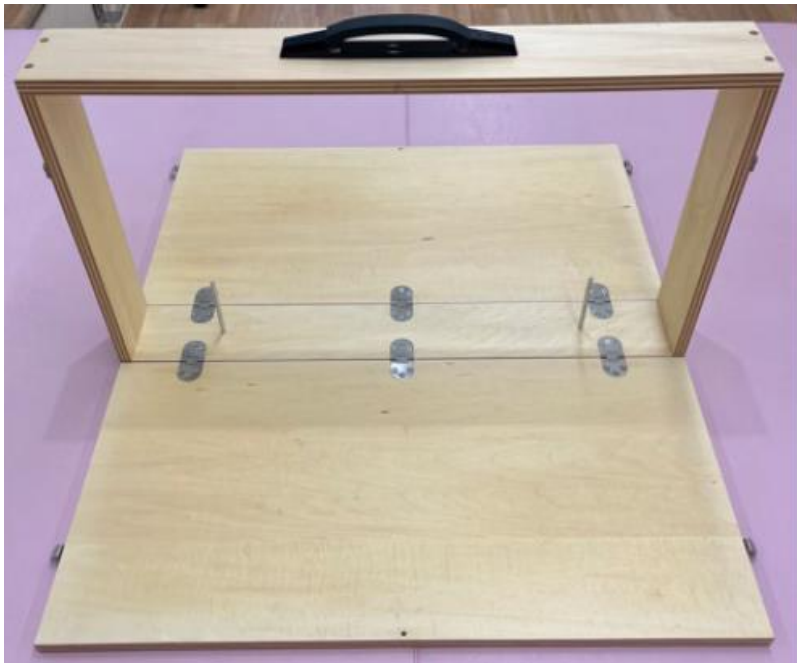

(d)

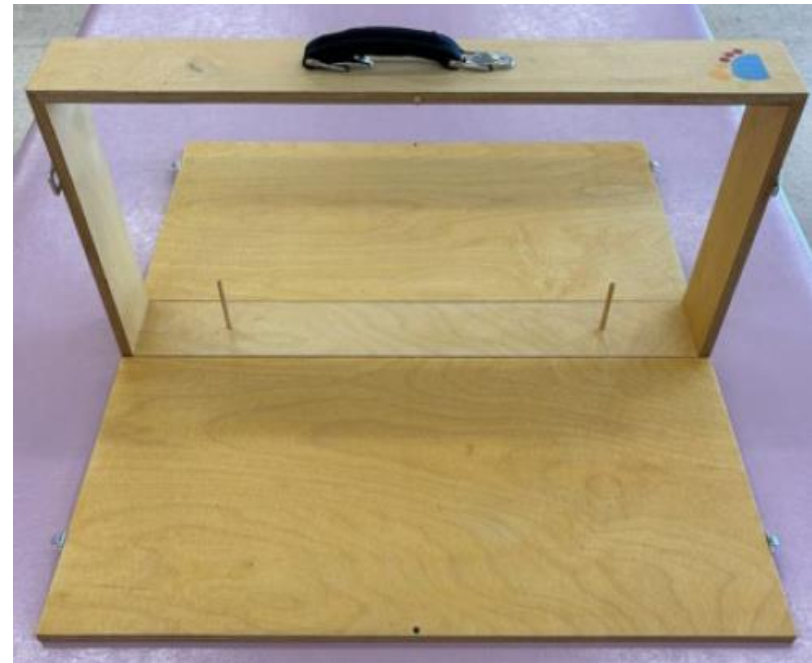

Supplement: S4 Appendix — (a) New ARAT equipment, (b) Conventional ARAT equipment, (c) New ARAT Platform, (d) Conventional ARAT Platform. ARAT, Action Research Arm Test. (PDF) [file pone.0334199.s004.pdf]
