# Supplementary material for: Reliability and validity of a newly developed Action Research Arm Test for upper limb function assessment in patients with stroke: A comparison with the conventional version
Source: PLoS One. 2026 Mar 24;21(3):e0334199. doi: 10.1371/journal.pone.0334199 (PMC13012481; doi:10.1371/journal.pone.0334199)
Supplement: S5 Table — (DOCX) [file pone.0334199.s005.docx]

**S 5 Table. Specifications of the main equipment**

| **Subscale** | **No.** | **Item** | **Specification Item** | **Conventional ARAT** | **NEW ARAT** |
| --- | --- | --- | --- | --- | --- |
| Grasp | 1 | Block 10 cm | Dimensions | 100 × 100 × 100 mm | 100 × 100 × 100 mm |
|  |  |  | Weight | 734 g | 665 g |
|  |  |  | Material | Wood | Wood |
|  | 2 | Block 2.5 cm | Dimensions | 25 × 25 × 25 mm | 25 × 25 × 25 mm |
|  |  |  | Weight | 11 g | 11 g |
|  |  |  | Material | Wood | Wood |
|  | 3 | Block 5.0 cm | Dimensions | 50 × 50 × 50 mm | 50 × 50 × 50 mm |
|  |  |  | Weight | 89 g | 91 g |
|  |  |  | Material | Wood | Wood |
|  | 4 | Block 7.5 cm | Dimension | 75 × 75 × 75 mm | 75 × 75 × 75 mm |
|  |  |  | Weight | 292 g | 288 g |
|  |  |  | Material | Wood | Wood |
|  | 5 | Cricket ball | Diameter | 68 mm | 68 mm |
|  |  |  | Weight | 123 g | 105 g |
|  |  |  | Material | Wood | Wood |
|  | 6 | Sharpening stone | Diameter | 99 × 10.5 × 25 mm | 100.5 × 10.3 × 25 mm |
|  |  |  | Weight | 72 g | 76 g |
|  |  |  | Material | Ceramic | Ceramic |
| Grip | 7 | Glass | Height | 129.6 mm | 129.5 mm |
|  |  |  | Diameter (upper, lower) | 72.1mm, 54.9 mm | 74.5 mm, 57.0 mm |
|  |  |  | Weight | 37 g | 58 g |
|  |  |  | Material | Polypropylene | Acrylonitrile-Styrene resin |
|  | 8 | Tube 2.25 cm | Length | 115 mm | 114 mm |
|  |  |  | Diameter (outer, inner) | 25 mm, 21 mm | 25 mm, 21 mm |
|  |  |  | Weight | 44 g | 44 g |
|  |  |  | Material | Aluminium | Aluminium |
|  | 9 | Tube 1.0 cm | Length | 160 mm | 159 mm |
|  |  |  | Diameter (outer, inner) | 10 mm, 7 mm | 10 mm, 7 mm |
|  |  |  | Weight | 17 g | 18 g |
|  |  |  | Material | Aluminium | Aluminium |
|  | 10 | Washer | Diameter (outer, inner) | 35 mm, 7 mm | 35 mm, 12 mm |
|  |  |  | Thickness | 3 mm | 2.85 mm |
|  |  |  | Weight | 20 g | 19 g |
|  |  |  | Material | Iron | Iron |
| Pinch | 11, 13, 14 | Ball bearing | Diameter | 6 mm | 6 mm |
|  |  |  | Weight | 1 g | 1 g |
|  |  |  | Material | Iron | Iron |
|  | 12. 15, 16 | Marble | Diameter | 15.8 mm | 14.5 mm |
|  |  |  | Weight | 5 g | 4 g |
|  |  |  | Material | Glass | Glass |

Dimensions are presented as width (W) × depth (D) × height (H).

ARAT, Action Research Arm Test.
