# Supplementary material for: Reliability and validity of a newly developed Action Research Arm Test for upper limb function assessment in patients with stroke: A comparison with the conventional version
Source: PLoS One. 2026 Mar 24;21(3):e0334199. doi: 10.1371/journal.pone.0334199 (PMC13012481; doi:10.1371/journal.pone.0334199)
Supplement: S6 Table — (DOCX) [file pone.0334199.s006.docx]

**S 6 Table. Specifications of the auxiliary equipment**

| **Subscale used in** | **Name** | **Item** | **Specification Item** | **Conventional ARAT** | **NEW ARAT** |
| --- | --- | --- | --- | --- | --- |
| Grip | Stand (large) |  | Weight | 1442 g | 1564 g |
|  |  |  | Material | Wood | Wood |
|  |  | Base | Dimensions | 440 × 100 × 50 mm | 440 × 100 × 50 mm |
|  |  | Rod (thick) | Diameter | 20 mm | 20 mm |
|  |  |  | Height (from base) |  |  |
|  |  | Rod (thin) | Diameter | 6 mm | 6 mm |
|  |  |  | Height (from base) |  |  |
| Grip | Stand (small) |  | Weight | 127 g | 140 g |
|  |  |  | Material | Wood | Wood |
|  |  | Base | Dimensions | 200 × 50 × 15 mm | 200 × 50 × 15 mm |
|  |  | Rod (thick) | Diameter | 20 mm | 20 mm |
|  |  |  | Height (from base) |  |  |
|  |  | Rod (thin) | Diameter | 6 mm | 6 mm |
|  |  |  | Height (from base) |  |  |
| Grip | Rod |  | Diameter | 6 mm | 6 mm |
|  |  |  | Height | 80 mm | 82 mm |
|  |  |  | Material | Aluminium | Aluminium |
| Pinch | Case |  | Weight | 60 g | 34 g |
|  |  |  | Material | Aluminium | Aluminium |
|  |  | Lid | Height | 10 mm | 14 mm |
|  |  |  | Depth | 9.6 mm | 9.5 mm |
|  |  |  | Diameter | 105 mm | 85 mm |
|  |  | Base | Height | 26 mm | 25 mm |
|  |  |  | Diameter | 100 mm | 80 mm |
|  |  |  | Weight | 123 g | 105 g |
| Grasp, Pinch | Platform (unfolded) |  | Dimensions | 720 × 845 × 380 mm | 720 × 843 × 380 mm |
|  |  |  | Weight | 12.06 kg | 11.15 kg |
|  |  |  | Material | Wood | Wood |

Dimensions are presented as width (W) × depth (D) × height (H). Stand consists of a rod and a base.

ARAT, Action Research Arm Test.
