# Supplementary material for: Reliability and validity of a newly developed Action Research Arm Test for upper limb function assessment in patients with stroke: A comparison with the conventional version
Source: PLoS One. 2026 Mar 24;21(3):e0334199. doi: 10.1371/journal.pone.0334199 (PMC13012481; doi:10.1371/journal.pone.0334199)
Supplement: S7 Table — (DOCX) [file pone.0334199.s007.docx]

**S 7 Table. Correlations between the Action Research Arm Test total score and clinical measures in the reliability and validity testing groups**

| **Variable** | **Reliability testing group (n = 33)** | | **Validity testing group (n = 31)** | |
| --- | --- | --- | --- | --- |
|  | **Spearman’s ρ** | ***p*** | **Spearman’s ρ** | ***p*** |
| FMA-UE total | 0.924 | <0.001 | 0.890 | <0.001 |
| BBT paretic side | 0.929 | <0.001 | 0.926 | <0.001 |
| Grip strength (kg) paretic side | 0.606 | <0.001 | 0.780 | <0.001 |
| MAL Amount of Use | 0.718 | <0.001 | 0.455 | 0.010 |
| MAL Quality of Movement | 0.668 | <0.001 | 0.622 | <0.001 |
| JASMID Quantity | 0.788 | <0.001 | 0.762 | <0.001 |
| JASMID Quality | 0.851 | <0.001 | 0.875 | <0.001 |

The strength of the correlation was interpreted based on the absolute value of the correlation coefficient as follows: <0.4 = weak, 0.4–0.74 = moderate, 0.75–0.9 = strong, and ≥0.9 = very strong. 　　　　　　　　　　　　　　　　　　　　　ARAT, Action Research Arm Test; BBT, Box and Block Test; FMA-UE, Fugl-Meyer Assessment of the Upper Extremity; JASMID, Jikei Assessment Scale for Motor Impairment in Daily Living; MAL, Motor Activity Log.
