# Supplementary material for: Reliability and validity of a newly developed Action Research Arm Test for upper limb function assessment in patients with stroke: A comparison with the conventional version
Source: PLoS One. 2026 Mar 24;21(3):e0334199. doi: 10.1371/journal.pone.0334199 (PMC13012481; doi:10.1371/journal.pone.0334199)
Supplement: Attachment — Submitted filename: 20260122_Response_reviewer_2_arat.docx [file pone.0334199.s010.docx]

**RESPONSES TO REVIEWER COMMENTS**

**Reviewer #2**

**I laud the authors on their well-crafted manuscript of an expertly designed and responsibly executed study! Please see the attached .pdf file for my detailed commentary (which I hope that the authors will be capable of addressing).**

We sincerely appreciate your insightful comments and suggestions, which have helped enhance the manuscript and provide a more balanced and comprehensive account of our research. We have addressed all your comments below and incorporated the missing elements into the revised manuscript.

**Comment 1**

**Please explain overlaps in kappa coefficient scale.**

Response:

We appreciate your insightful comments. We have revised the presentation of the weighted kappa coefficient scale throughout the manuscript for clarity and consistency.

Introduction, page 11, lines 228-230:

“*The weighted kappa coefficients were interpreted as follows [32]: <0.00 = poor, 0.00–0.20 = slight, 0.21–0.40 = fair, 0.41–0.60 = moderate, 0.61–0.80 = substantial, and 0.81–1.00 = almost perfect.*”

Results, Table 4, page 17, line 314:

“*The weighted kappa coefficients were interpreted as follows: <0.00 = poor, 0.00–0.20 = slight, 0.21–0.40 = fair, 0.41–0.60 = moderate, 0.61–0.80 = substantial, and 0.81–1.00 = almost perfect.*”

Results, Table 5, page 19, line 317:

“*The weighted kappa coefficients were interpreted as follows: <0.00 = poor, 0.00–0.20 = slight, 0.21–0.40 = fair, 0.41–0.60 = moderate, 0.61–0.80 = substantial, and 0.81–1.00 = almost perfect.*”

**Comment 2**

**Please speculate on why the bias values were so abnormally high for the inter-rater assessments of new *vs* original ARAT; could this, at all, be related to the variations in material compositions, object dimensions, and/or surface textures between the two versions of the ARAT?**

Response:

Thank you for your insightful comments. We investigated how differences in the physical properties of test items in the new ARAT influenced task difficulty and patients’ motor strategies, which likely contributed to the relatively larger bias observed in the Grasp item when comparing the two versions.

Results, page 20, lines 320-325:

“*Mean bias values were generally minimal across subscales and total scores (range: –0.091 to 0.303 points). Notably, the Grasp subscale in the inter-rater comparison showed a relatively higher bias (0.303) compared with other subscales (range: –0.091 to 0.030), although this value remained within clinically acceptable limits. Despite this finding, the 95% limits of agreement for all measures, including Grasp, fell within the clinically acceptable range defined by MDC values, confirming measurement equivalence between the two versions.*”

Discussion, page 23, lines 407-423:
“*Although the new ARAT maintained identical assessment protocols and scoring criteria compared with the conventional version, some test objects were modified in size and materials. These changes were implemented to ensure cost-effective manufacturing and long-term stable supply, which are essential for sustained clinical availability in Japan after discontinuing the conventional ARAT import. The Bland–Altman analysis revealed that the Grasp subscale in the inter-rater comparison exhibited a relatively higher bias (0.303) than other subscales. The observed bias in Grasp tasks likely reflects the sensitivity of grasping movements to subtle differences in test object characteristics between the new and conventional ARAT versions. Among the modified objects, grasp-related items showed the greatest variations in dimensions (2–5 mm; see S2 and S3 Tables) and surface textures, which may have influenced tactile feedback, grip stability, and perceived task difficulty. These physical differences could affect patients' motor execution strategies and evaluators' scoring judgments, resulting in the observed systematic difference. However, this bias remained within clinically acceptable limits, as the LOA fell within the MDC₉₅ range, indicating preserved measurement equivalence with no meaningful impact on decision-making. Overall, the minor equipment modifications required for domestic manufacturing and stable supply did not substantially affect the measurement properties that have been maintained for decades with the conventional ARAT.*”

**Comment 3**

**Please attend to my commentary to the left of Figure 4.**

**These scatterplots (putatively) appear to be either bimodal or trimodal. 0. Would it be clinically appropriate to stratify the data before conducting stratified correlation analyses? 1. Would the authors please A. discuss the different the clinical characteristics of the patient populations that populate each sector (***various machine learning-based clustering analyses would seem appropriate***) and B. comment on the implications of the assessment results for the personalization of their stroke neurorehabiblitation regimens?**

Response:

We sincerely appreciate your important comments and have carefully considered the points you raised. As the reviewer noted, Figures 4 and 5 highlight distribution patterns specific to certain data, and stratification or machine learning-based clustering analyses could be useful approaches. However, given that these analyses would deviate from the primary objective of this study, the limited sample size, and the absence of predefined stratification criteria, conducting exploratory machine learning analyses at this stage carries a high risk of overfitting and potential overinterpretation. Therefore, these methods were not applied in this study. Nevertheless, the observed distribution characteristics suggest that ARAT scores reflect multidimensional aspects of upper limb function, and we have added these points to the Discussion section.

Discussion, page 24, lines 442-450:

“*Scatter plots presented in Figures 4 and 5 revealed clustering patterns in which patients with similar ARAT scores exhibited substantial variability in other clinical measures, and vice versa. This heterogeneity was particularly evident in the low-score range (ARAT < 10) and the high-score range (ARAT > 50), where patients clustered at discrete ARAT values yet showed wide dispersion in FMA-UE, BBT, and self-reported outcomes. These patterns suggest that ARAT scores reflect multidimensional aspects of upper limb function, and that patients achieving comparable ARAT performance may differ considerably in underlying motor capacity (FMA-UE), manual dexterity (BBT), maximal force generation (grip strength), and perceived functional use (MAL/JASMID).*”

**Comment 4**

**Considering that grip strength assessment is a known predictor of patient prognosis following an acute traumatic event such as a stroke or a fall, could the authors please comment on whether they think that a combined assessment of *ARAT + grip strength* would be prognostically superior to ARAT alone.**

Response:

We appreciate the reviewer's insightful comments. The combined assessment of ARAT and grip strength is expected to provide complementary information, as each measure reflects distinct aspects of upper limb function, allowing for a more comprehensive evaluation. In response, we have added a reference to the relevant literature and expanded the Discussion section accordingly.

Discussion, page 24, lines 428-436:

“*Grip strength is a known predictor of functional prognosis after stroke [41,42], and previous studies have reported that a combined assessment of ARAT grasp performance and grip strength is useful for predicting upper limb function outcomes [43]. This predictive value is presumed to derive from the complementary information provided by the two measures: whereas the ARAT grasp task (ρ = 0.683) evaluates functional object manipulation under standardized conditions, grip strength quantifies maximal voluntary muscle force. The moderate correlation observed in this study indicates that these measures assess related but distinct dimensions of upper limb function, supporting their combined use for comprehensive clinical evaluation and individualized goal setting.*”

“♯Reference 41. Suzuki M, Sugimura S, Suzuki T, Sasaki S, Abe N, Tokito T, et al. Machine-learning prediction of self-care activity by grip strengths of both hands in poststroke hemiplegia. Medicine (Baltimore). 2020;99: e19512. doi: 10.1097/MD.0000000000019512.”

“♯Reference 42. Snickars J, Persson HC, Sunnerhagen KS. Early clinical predictors of motor function in the upper extremity one month post-stroke. J Rehabil Med. 2017;49: 216-222. doi: 10.2340/16501977-2205.”

“♯Reference 43. Alt Murphy M, Al-Shallawi A, Sunnerhagen KS, Pandyan A. Early prediction of upper limb functioning after stroke using clinical bedside assessments: a prospective longitudinal study. Sci Rep. 2022;12: 22053. doi: 10.1038/s41598-022-26585-1.”

**Comment 5**

**Although the authors' rationale is well-taken, please analyze the reliability and the validity testing groups individually and report in supplementary materials those best practices-adherent results.**

Response:

Thank you very much for your important comments. We conducted correlation analyses separately by group, and the results are presented in Supporting Information 4 (S4 Table).

Materials and Methods, Instruments, Statistical Analysis, page 11, lines 245-246:

“*Furthermore, correlation analyses were conducted separately for the reliability testing group and the validity testing group.*”

Results, page 21, lines 368-369:

“*The results of the group-specific correlation analyses are presented in S4 Table.*”

Supporting information, page 32, lines 650-651:

“***S4 Table.*** *Correlations between the Action Research Arm Test total score and clinical measures in the reliability and validity testing groups*”

We sincerely thank the reviewers for their valuable comments and suggestions, which have greatly contributed to improving our manuscript.
